# Retrograde Tibial Nailing: a minimally invasive and biomechanically superior alternative to angle-stable plate osteosynthesis in distal tibia fractures

**DOI:** 10.1186/1749-799X-9-35

**Published:** 2014-05-13

**Authors:** Sebastian Kuhn, Philipp Appelmann, Dorothea Mehler, Philip Pairon, Pol M Rommens

**Affiliations:** 1 Department of Orthopedics and Traumatology, University Medical Centre of the Johannes Gutenberg University, Langenbeckstrasse 1, Mainz 55131, Germany

**Keywords:** Distal tibia, Metaphyseal fractures, Intramedullary nailing, Retrograde nailing, Plate osteosynthesis

## Abstract

**Background:**

Currently, antegrade intramedullary nailing and minimally invasive plate osteosynthesis (MIPO) represent the main surgical alternatives in distal tibial fractures. However, neither choice is optimal for all bony and soft tissue injuries. The Retrograde Tibial Nail (RTN) is a small-caliber prototype implant, which is introduced through a 2-cm-long incision at the tip of the medial malleolus with stab incisions sufficient for interlocking. During this project, we investigated the feasibility of retrograde tibial nailing in a cadaver model and conducted biomechanical testing.

**Methods:**

Anatomical implantations of the RTN were carried out in AO/OTA 43 A1-3 fracture types in three cadaveric lower limbs. Biomechanical testing was conducted in an AO/OTA 43 A3 fracture model for extra-axial compression, torsion, and destructive extra-axial compression. Sixteen composite tibiae were used to compare the RTN against an angle-stable plate osteosynthesis (Medial Distal Tibial Plate, Synthes^®^). Statistical analysis was performed by Student's *t* test.

**Results:**

Retrograde intramedullary nailing is feasible in simple fracture types by closed manual reduction and percutaneous reduction forceps, while in highly comminuted fractures, the use of a large distractor can aid the reduction. Biomechanical testing shows a statistically superior stability (*p* < 0.001) of the RTN during non-destructive axial loading and torsion. Destructive extra-axial compression testing resulted in failure of all plate constructs, while all RTN specimens survived the maximal load of 1,200 N.

**Conclusions:**

The prototype retrograde tibial nail meets the requirements of maximum soft tissue protection by a minimally invasive surgical approach with the ability of secure fracture fixation by multiple locking options. Retrograde tibial nailing with the RTN is a promising concept in the treatment of distal tibia fractures.

## Background

Distal tibia fractures show a bimodal epidemiologic distribution with a peak in young males and elderly females [1,2]. In young patients, high-energy trauma often leads to associate soft tissue damage, while the elderly patients often display osteopenia and pre-existing soft tissue and/or vascular compromise [1,2]. Due to the demographic changes in western societies, the average age of patients with distal tibia fractures is rising. AO/OTA 43 A2/3 fractures can be considered as fragility fractures with an average patient age of almost 65 years [3].

Careful soft tissue handling is as relevant as the treatment of the bony injury itself. It is crucial to spare the extra-osseous vessels during surgery as best as possible [4]. Pre-existing injury- and surgery-related factors all contribute to disturbed healing and increase the risk of cortical necrosis, delayed or non-union, or infection [4,5]. Given these anatomical considerations, a minimally invasive surgical approach for the stabilization of distal tibia fractures is desirable since it reduces the risks of soft tissue and bone healing disturbances. Minimally invasive plate osteosynthesis (MIPO) has attracted great interest in the management of distal tibia fractures [6]. It is often promoted for being minimally invasive with implants being low profile and anatomically pre-contoured. Clinical series, however, report a high incidence of implant prominence and soft tissue irritation between 32% and 52% [7,8]. As an alternative technique, intramedullary nailing not only features favorable mechanical properties but also biological advantages due to the preservation of the vascularity of the fracture site and the integrity of the surrounding soft tissues. However, antegrade intramedullary nailing of far distal tibia fractures is challenging and bears the risk of primary and secondary malalignment. Recent literature shows that the optimal method of treatment in distal tibia fractures remains debatable [9-11].

Retrograde intramedullary nailing of tibial fractures has not been introduced in routine clinical practice and only a few selected cases have been reported in literature [12-15]. This is mainly due to the lack of an adequately designed implant. Given an adequate implant, a closed surgical approach by retrograde tibial nailing seems an attractive option since it could offer stable fracture fixation with minimal additional soft-tissue injury and sparing of the knee. The retrograde tibial nail (RTN) is an experimental intramedullary implant which has demonstrated favorable biomechanical properties in comparison to antegrade nailing [16,17].

In this project, we investigated if the RTN meets the requirements of a secure fracture fixation and a minimally invasive surgical approach. The hypothesis for the implantation in anatomical specimen was that retrograde intramedullary nailing is feasible with the newly developed RTN. The hypothesis for comparative biomechanical testing with an angle-stable plate osteosynthesis was that the RTN provides superior biomechanical properties in respect to extra-axial compression, torsion, and load to failure testing in an extra-articular distal tibia fracture model.

## Methods

### Anatomical insertion study

For the surgical feasibility study of the RTN, three fresh cadaveric lower extremities with no previous medical history related to the investigated limbs were obtained from the Anatomical Institute of our University. The use of human cadaveric specimen was approved by the local ethics committee (reference number 837.088.07, 28 March 2007). The RTN is an experimental intramedullary nail under design by our research group. The nailing system features double proximal and triple distal locking options. The nail of 8 mm in diameter and 120 mm in length was implanted into three intact human lower limbs including all soft tissues. Three different fracture types (AO/OTA 43 A1-3) were simulated by osteotomy via a posterolateral approach. After reduction, a 2–2.5-cm-long skin incision was started 1 cm proximally to the tip of the medial malleolus and was extended distally. The tip of the malleolus was marked with a Kirschner wire under fluoroscopic control in anteroposterior (a.p.) and lateral view. The wire was drilled parallel to the medial cortex at a distance of approximately 5 mm. The cortex of the malleolus was opened and a path was created by a sharp 9-mm cannulated awl. The awl was advanced with low force and twisting motions until the medullary canal was reached. The awl and wire were then removed. The nail, assembled on the aiming device, was introduced with low force and small twisting movements until its end sat flush with the entry portal. The nail position was confirmed in a.p. and lateral fluoroscopic views. The trocar combination was inserted until it touched the bony surface and the trocar was removed. Drilling was performed with a 3.2-mm drill bit to the desired depth. In all positions except for the second most distal locking position, which directs at the distal tibiofibular joint, bicortical drilling was carried out. Distal locking was achieved with 4.0-mm dual-core screws, specially designed for optimized purchase in cancellous bone. Proximally, the RTN was locked with standard 4.0-mm cortical screws. Finally, an end cap was introduced at the nail end to create a fixed-angle construct with the most distal screw. All implantations were carried out by the same orthopedic surgeon.

### Biomechanical testing

We conducted biomechanical testing to investigate the properties of the two implant types. Our null hypothesis was that there is no difference in the biomechanical properties after RTN osteosynthesis compared to angle-stable plate osteosynthesis (Medial Distal Tibial Plate, Synthes^®^, West Chester, PA, USA) in an extra-articular distal tibial fracture model (AO/OTA 43 A3) [18]. Fourth-generation biomechanical composite bone tibiae (Sawbones Europe, Malmö, Sweden, item number 3401) were used for the experiments. Based on *a priori* power analysis, 16 composite tibiae, 8 in each group, were tested in the comparative study. Implantations of the four-hole Medial Distal Tibial Plate (MDTP) were performed as stated in the manufacturer's instructions using three proximal and four distal screws. First, non-angular stable screws were used to fix the plate to the tibia proximally and distally. Afterwards, the two angle-stable screws were drilled and introduced proximally and three distally. The RTN was implanted as described above. All implantations were carried out by the same orthopedic surgeon.

After implantation into the intact composite bone, the osteotomy parameters were established. A transverse defect osteotomy of 10 mm was performed to simulate an AO/OTA 43 A3 type fracture. The bone between 40 and 50 mm from the tibial plafond was resected by a parallel saw. The proximal and distal segments were potted in poly-methyl methacrylate (PMMA) with two screws at each interface to provide a secure force transmission. To ensure that no direct contact between the PMMA and implants existed, all protruding parts were covered with modeling clay prior to potting. Additionally, all samples underwent radiological control in a.p. and lateral view to exclude possible damages and to document the correct implantation.

A universal pneumatic testing machine (SincoTec Test Systems, Clausthal-Zellerfeld, Germany) controlled by PneuSys software (SincoTec, Clausthal-Zellerfeld, Germany) was used for testing. The setup was based on the method described by Hansen et al. [19]. For extra-axial compression, the point of load transmission was in accordance to Horvitz et al. [20]. The force was applied to the tibial plateau at a 10-mm posteromedial offset from the tibial eminentia. A steel ball in a trough coupled with a force-transmitting bar applied the pressure to the plateau. Distally, a cardan joint was used to position the samples in the testing machine since the human ankle joint is best simulated by this method.

The axial loading began under a constant preload of 18 N and was increased to 350 N before decreasing back to 18 N. Each cycle had duration of 20 s. After a pre-cycle, three measurement cycles were recorded. During that phase, the actuator connected to the force-transmitting bar of the testing machine recorded axial movements.

For torsional testing, the specimens were anchored at both ends. Bidirectional torque was applied under a constant preload of 10 N. A cycle consisted of torsion from 0 to 8 Nm clockwise and back to 8 Nm counter-clockwise with duration of 20 s per cycle. One pre-cycle and three measurement cycles were conducted in this study. During that time a rotational transducer connected to the actuator of the testing machine recorded the rotational difference of the proximal to the distal segment.

As a final investigation, an axial ‘load-to-failure’ test was performed for extra-axial compression of up to 1,200 N.

Construct stiffness was measured during axial loading tests by the load to deformation curve. Rotational stiffness was measured by the torsion to rotation curve. The correlation of the constructs' linear-elastic area of the force-displacement and force-angle diagram was the basis for the non-destructive test evaluations. Force-failure values were recorded and analyzed for load-to-failure test. Failure was defined as plastic deformation of the construct. All constructs underwent the identical testing sequence. First, a non-destructive test for extra-axial force with 350 N was performed. Afterwards, a bidirectional rotational test with 8 Nm was done. Finally, increased loading for destructive loading tests took place for extra-axial compression up to 1,200 N. Table 1 gives an overview of the testing sequence.

**Table 1:** Test sequence of the biomechanical evaluation

**Test sequence**	**8 RTN vs. 8 MDTP**	
Non-destructive tests	Extra-axial compression, 350 N	Torsion, 8 Nm
Destructive tests	Extra-axial compression up to 1,200 N	

### Statistics

An *a priori* power analysis was performed to determine the sample size (eight in each group) for the biomechanical testing. Statistical analysis was performed with SPSS software (SPSS 21.0, IBM, Armonk, NY, USA). The force-displacement and force-angle values of the two implant types were compared with the Student's *t* test. An error level of less than 5% (*p* value, 0.05) was defined as statistically significant.

## Results

### Anatomical insertion study

**Figure 1:**
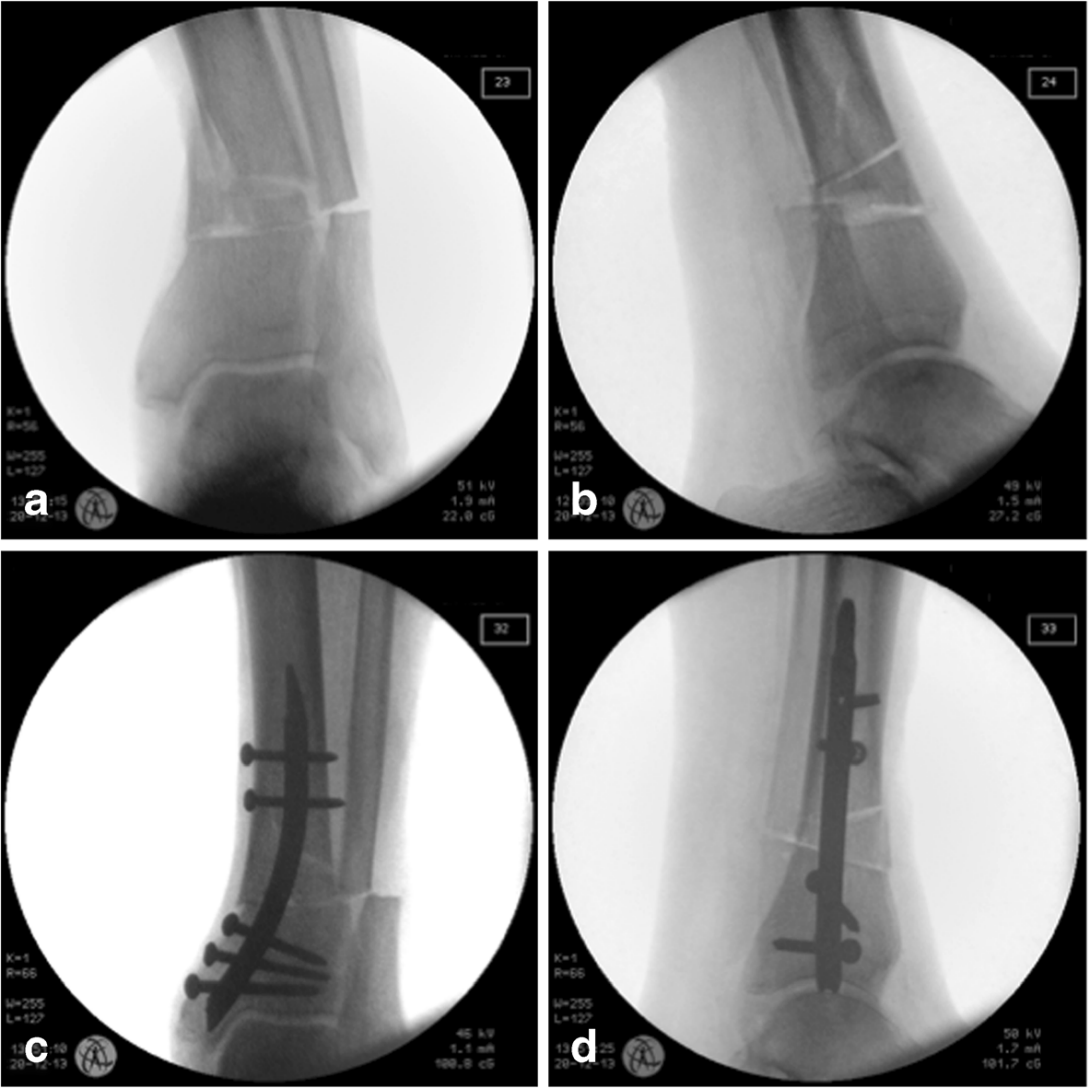
**Implantation of RTN in an AO/OTA 43 A3 fracture.** Implantation of RTN in a cadaveric lower limb with an AO/OTA 43 A3 fracture. Fracture pattern in **(a)** anterioposterior and in **(b)** lateral view. The convergence of the locking screws towards the plafond in a.p. **(c)** and the deviation in lateral view **(d)**.

**Figure 2:**
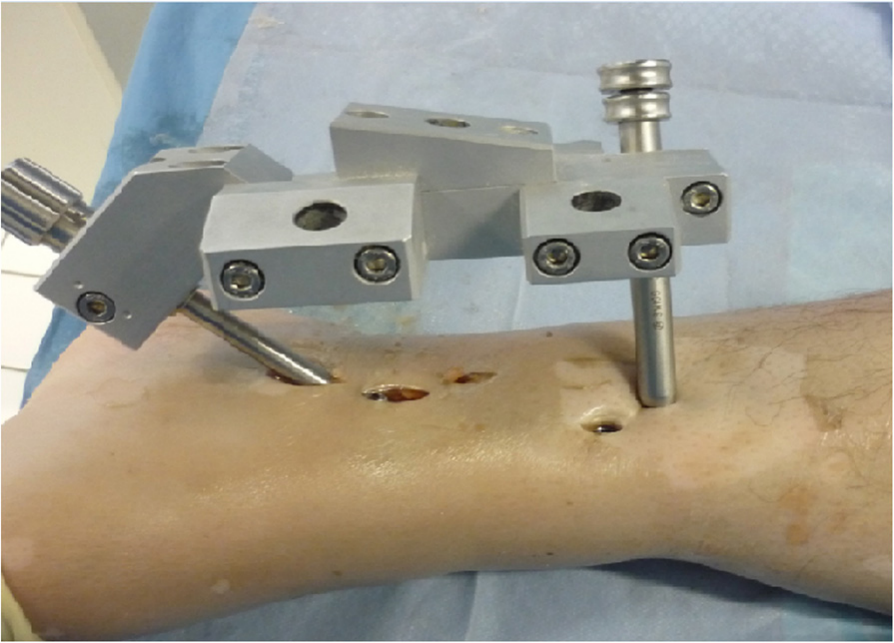
**Minimally invasive surgical implantation of the RTN.** Osteosynthesis with the RTN is possible through limited skin incisions. Human cadaver specimen of a left lower limb. The RTN is inserted through the medial malleolus, while interlocking is performed though separate small incisions. The aiming device allows for x-ray free interlocking at all levels.

Retrograde intramedullary nailing with the newly developed RTN was possible in a lab setting without major complication through limited skin incisions of 2–2.5 cm for the nail insertion and stab incisions for interlocking. When the entry portal is chosen on the medial surface of tip of the medial malleolus and the trajectory parallel to the medial cortex, the risk of damaging or fracturing the medial malleolus is very low. In our study, this did not happen but it is a complication which has to be taken into account. Moreover, no iatrogenic posterior tibial tendon injury occurred. In simple fracture types, closed manual reduction with the aid of percutaneous reduction forceps is possible. In highly comminuted fractures like the AO/OTA 43 A3, the use of a temporary large distractor may be useful to achieve correct axis and rotation prior to intramedullary nailing. Otherwise, primary malalignment can result. Figure 1 shows an implanted RTN in a cadaveric lower limb with an AO/OTA 43 A3 fracture. Figure 2 demonstrates the limited skin incisions that are necessary for implantation of the RTN.

### Biomechanical testing

#### Non-destructive extra-axial compression

**Figure 3:**
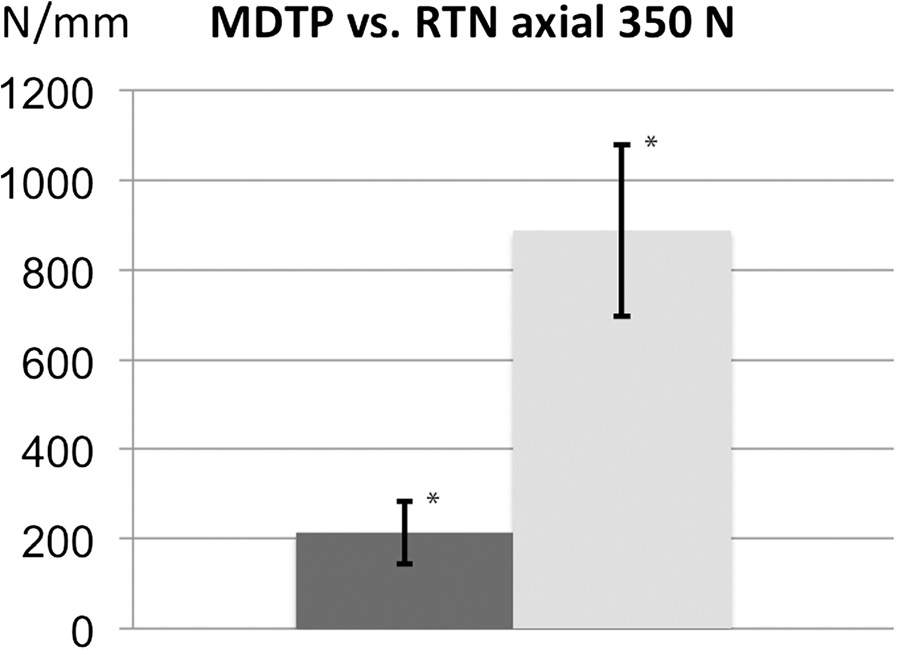
**Results of the axial stability testing.** Axial construct stability for extra-axial force with 350 N for the MDTP (dark) and RTN (light). Results show statistically significant differences (*p < 0.05) with higher stability for the RTN.

Non-destructive axial compression testing resulted in higher stability for the RTN in comparison to the medial distal tibial plate (RTN 888 ± 192 N/mm vs. MDTP 213 ± 70 N/mm). Statistical analysis proved a significant difference between the RTN and MDTP group (*p* < 0.001). Results are shown in Figure 3.

### Torsion

**Figure 4:**
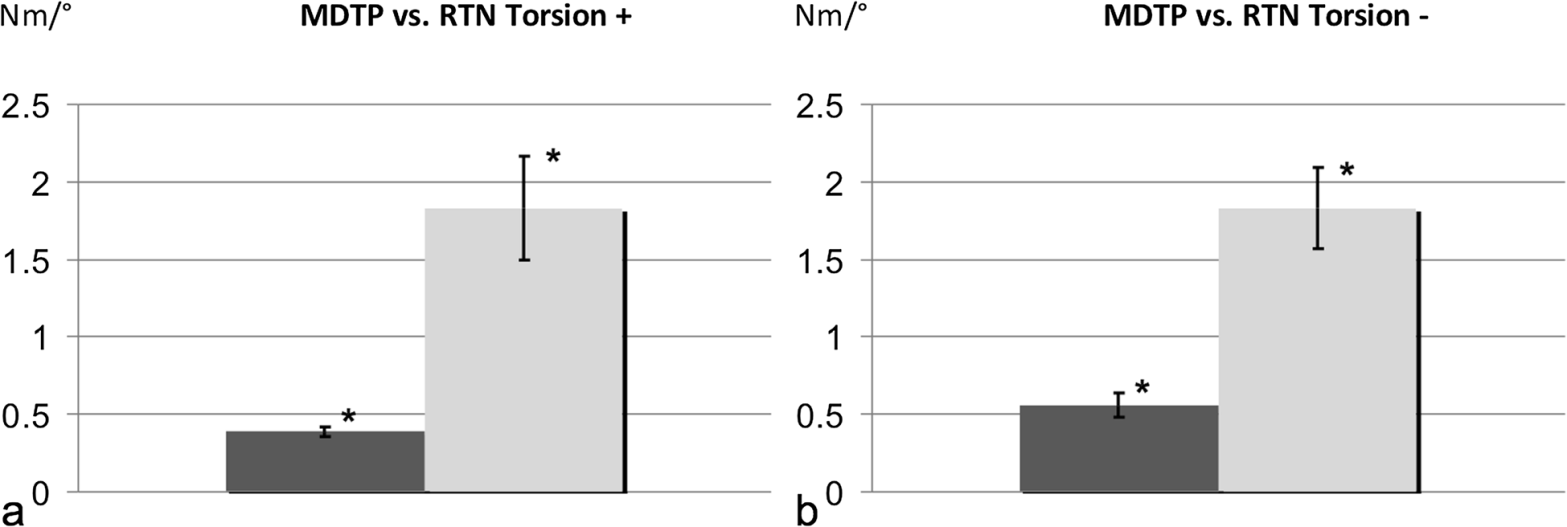
**Results of the torsional stability testing.** Torsional stability testing for the MDTP (dark) and RTN (light). Results show statistically significant differences (*p < 0.05) with higher stability for the RTN in comparison to the MDTP in clockwise **(a)** and counter-clockwise **(b)** torsion.

Results show a higher stability for the RTN during clockwise and counter-clockwise rotations. The average force-angle value was 1.83 ± 0.33 Nm/° in the RTN vs. 0.39 ± 0.03 Nm/° in the MDTP group during clockwise rotation (Figure 4a) respectively, 1.83 ± 0.26 Nm/° in the RTN vs. 0.55 ± 0.08 Nm/° in the MDTP group during counter-clockwise rotation (Figure 4b). Statistical analysis proved a significant difference (*p* values < 0.001) between the RTN and MDTP group for torsional stability.

### Destructive extra-axial compression

**Figure 5:**
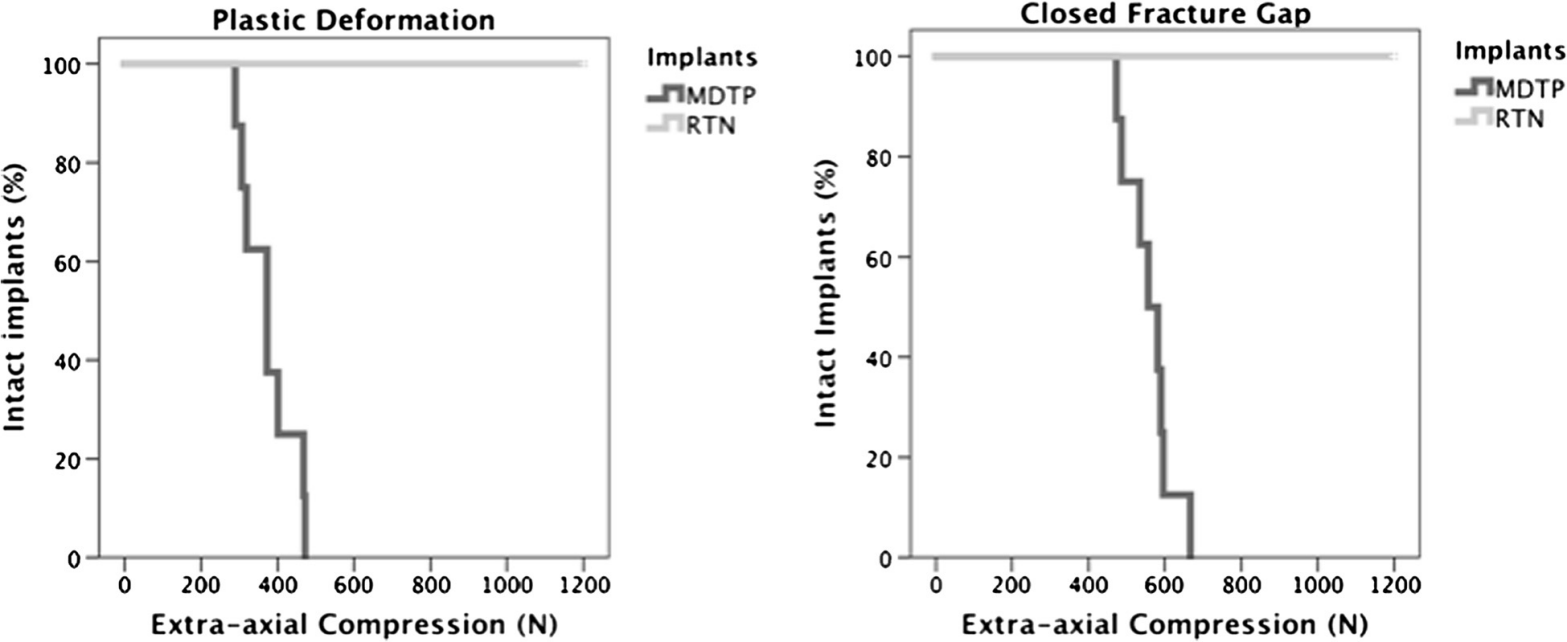
**Results of the destructive compression testing.** Kaplan-Meier survival curve for destructive extra-axial compression of up to 1,200 N for the MDTP (dark) and RTN (light). All RTN implants survived the maximal load of 1,200 N, while all MDTP showed failure in form of plastic deformation (left graph) and closure of the fracture gap (right graph).

**Figure 6:**
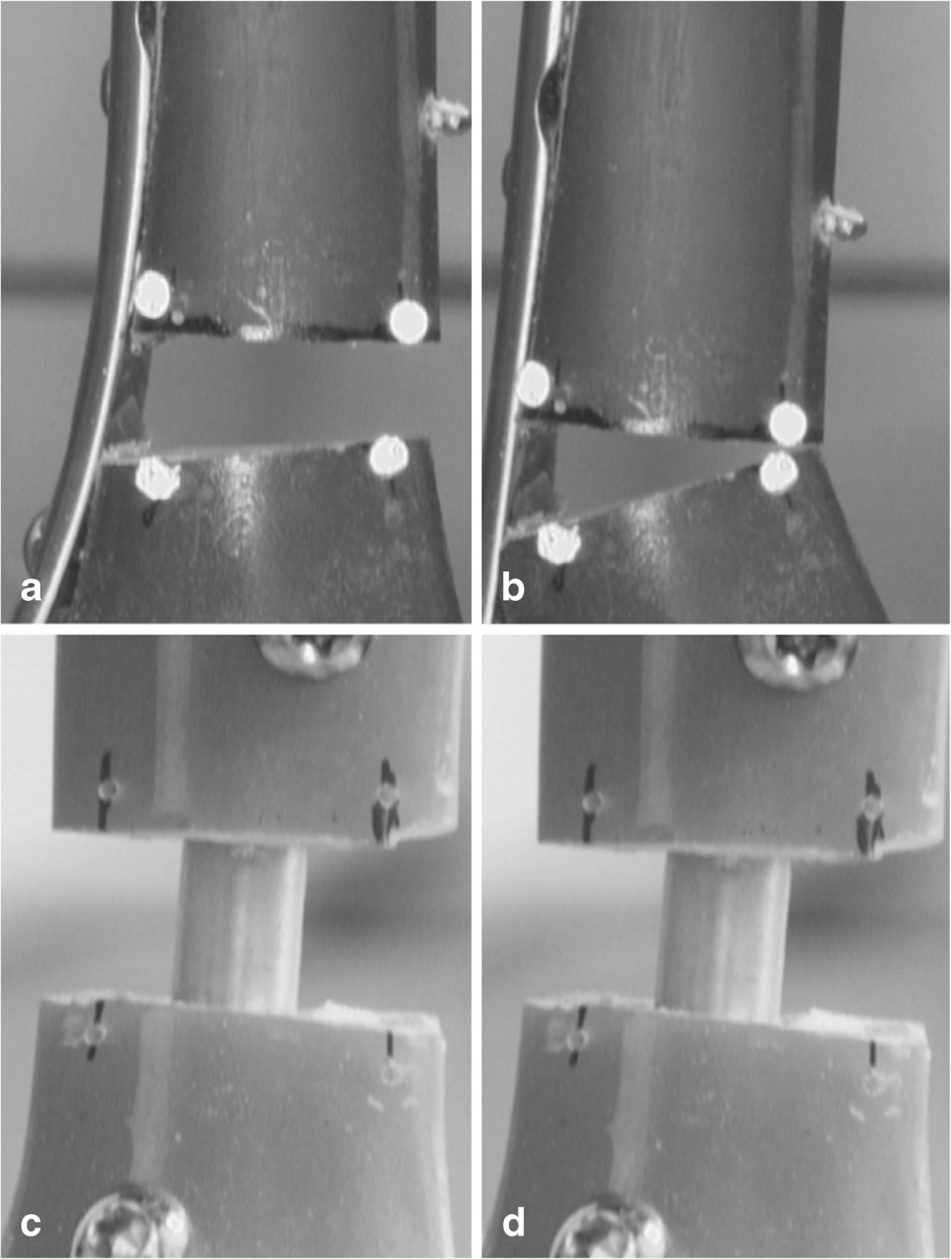
**Fracture gap under destructive compression testing.** Destructive extra-axial compression for the MDTP **(a, b)** and RTN **(c, d)** under preload of 18 N (a and c) and maximal extra-axial compression of up to 1,200 N (b and d). MDTP shows closure of the fracture gap **(b)**. All RTN implants withstand the maximal load of 1,200 N and show only minimal axial compression.

Destructive extra-axial compression resulted in failure of all MDTP constructs with an average value of 375 ± 73 N for plastic deformation and the fracture gap closed at an average value of 561 ± 63 N. Due to the extensive deformation, the load increase was stopped at 900 N in the MDTP group. The Kaplan-Meier survival curves for destructive extra-axial compression in the form of plastic deformation and closure of the fracture gap are shown in Figure 5. All RTN specimens survived the maximal load of 1,200 N, which was limited by the maximal force applied by the testing machine. Plastic deformation was not recorded in any RTN specimen. Images of the destructive extra-axial compression test are given in Figure 6.

## Discussion

The management of distal metaphyseal tibia fractures remains challenging and controversial [9]. Distal tibia fractures demand a stable fracture fixation while adding minimal additional trauma by the surgical approach and implants. Choosing an appropriate treatment can be difficult, especially in the elderly population, which make up the majority of patients. A combination of thin skin and compromised soft tissues and vascular blood supply are maybe present prior to the injury. An optimal treatment needs to take pre-existing soft tissue condition and the medical history of the patient into account. MIPO with the plate on the medial distal tibia is a reasonable choice but problematic in patients with poor soft tissues, peripheral arterial disease, or diabetes. MIPO has been advocated for reducing the injury to the soft tissues and producing good clinical results [11,21,22]. However, also in MIPO of distal tibia fractures, complications due to skin irritation from the plates, skin necrosis, and plate exposure have been published [8,23]. In our opinion, this specifically designed intramedullary implant for distal tibia fractures might provide advantages over the current choices. The skin incisions are limited to one 2-cm-long incision for nail entry and five 1-cm-long stab incisions for interlocking. While still located on the medial side, which is the usual site of the soft tissue injury, the RTN is less invasive than MIPO plating, which involves tunneling of the contused soft tissues.

The nail allows for stable fixation in the region of the distal 6 cm, which is currently problematic to treat. The unique RTN design allows addressing far distal tibial fractures. When the nail is placed optimally so that the most distal locking option runs just parallel to the plafond, all distal locking options are within 25 mm of the joint line. We mainly see indications for the RTN in far distal tibia shaft fractures (AO/OTA 42 A-C) and distal extra-articular metaphyseal tibia fractures (AO/OTA 43 A1-3).

As a conclusion from the surgical feasibility study, the critical points of the retrograde nailing technique in tibia fractures are selecting the right entry site and creating the correct path through the medial malleolus, avoiding any fissure or fracture. Applying this technique, no iatrogenic fracture occurred in our study. However, fracturing of the medial malleolus is a possible complication, which has to be taken into account, especially in elderly patients with osteoporotic bone quality.

As in other nailing procedures of metaphyseal lesions, the fracture must be reduced before nail insertion. Metaphyseal fractures will not be corrected by introducing the nail, whether it be antegrade or retrograde. If malalignment is present during nail insertion, the fracture will most likely be stabilized in a non-anatomical position [24,25]. Therefore, prior reduction and careful control of the fragments while introducing the nail across the fracture line are crucial. Failure to control the fragments will lead to primary malalignment.

An anatomical alignment may develop into secondary malalignment from displacement caused by relative instability between the bone and implant. In the distal fifth of the bone, the tibial cortex thins and the bone structure features a cancellous architecture. The spongiosa microstructure is dense in young patients but decreases with age [26]. Screw purchase, which allows good fixation in normal bone, is limited in osteoporotic bone [27]. Angular malunions have widely been reported in distal tibia fractures [28,29]. Prevention of secondary malalignment is largely dependent on biomechanical features of the implant and the implant to bone interface. In the presented biomechanical study, there are clear advantages of the RTN in comparison to the MDTP. Non-destructive axial compression and bidirectional torsion testing resulted in superior stability for the RTN. Destructive extra-axial compression resulted in failure of all MDTP constructs, while all RTN samples withstood the load-to failure test up to the maximal force applicable by the testing machine. This supports already published results where favorable biomechanical properties of the RTN were shown for torsion and load-to-failure test in comparison to antegrade nails [16,17]. In distal defect fractures, plates have an even low rigidity against axial compression, which are results of their geometry and excentric position [30].

In elderly patients, general weakness or dementia often prevents necessary non- or partial weight bearing on the injured leg. In these patients, the main treatment goal is restoration of function [31]. Nails are load-sharing while plates are load-bearing devices. Intramedullary nailing is the most efficient method to reduce strain at the bone-implant interface. In order to reduce stress-shielding in plates, long implants fixed with few locking head screws are currently used. However, it is less effective and leads to a relatively large surface implant. An intramedullary implant is in line with the weight-bearing axis while plates feature an excentric position. Earlier weight bearing might be possible after nail osteosynthesis. In elderly osteoporotic patients, fixing implants are usually not removed after fracture healing. Since metal is much more rigid than bone, there is a risk for stress rising at the plate end and bone absorption under the plate [32]. This causes additional structural bone weakening with an increased risk for a subsequent fracture. The use of a retrograde nail in combination with lag screw fixation might offer an option in certain pilon fractures (AO/OTA 43 C1-2), since in these patients, the main treatment goal should be the restoration of function over restoration of an exact anatomy. However, this needs to be further investigated.

There are some limitations to this study that we need to discuss, mainly, the use of artificial composite bones in biomechanical testing. Their stiff mechanical properties are similar to human bones. They represent a 183-cm tall, 90-kg heavy male with mechanical properties of healthy adult bones of less than 80 years [33]. In osteopenic/osteoporotic bones, failure will most likely occur at lower values. Nevertheless, biomechanical composite bones also display advantages and were therefore selected as a surrogate. There are no biological variations between samples, which might lessen the comparability between the specimens. Additionally, it has been proven that the failure modes and resulting fracture patterns are close to the ones published for human bones [33,34]. As with all uses of cadaveric implantations and biomechanical sawbone experiments in research, we recognize that our study can only provide an outlook of the later clinical performance of the implant.

## Conclusions

To conclude, the newly designed RTN combines the advantages of a small local, load-sharing, and minimally invasive implant with maximum soft tissue protection. The features include sparing of the knee and leaving the tibial isthmus uninvolved. Retrograde intramedullary nailing is feasible in far distal extra-articular distal tibia fractures. Biomechanical testing shows a statistically superior stability over angle-stable plate osteosynthesis. Therefore, we believe that the RTN concept provides both biological and mechanical advantages over extra-medullary systems like plates and could present a minimally invasive alternative to MIPO in distal extra-articular metaphyseal tibia fractures.

## Abbreviations

MDTP: medial distal tibial plate; MIPO: minimally invasive plate osteosynthesis; PMMA: poly-methyl methacrylate; RTN: retrograde tibial nail.

## Competing interests

The authors declare that they have no competing interests.

## Authors’ contributions

All authors made substantial contributions to this paper. The detailed parts of contributions were as follows: SK, PA, DM, PMR—study conception and design; SK, PA, DM, PP—acquisition of data; SK, PA, DM, PP, PMR—analysis and interpretation of data; SK—drafting of manuscript; SK, PA, DM, PP, PMR—critical revision. All authors gave final approval of the version submitted and agree to be accountable for all aspects of the work in ensuring that questions related to the accuracy or integrity of any part of the work are appropriately investigated and resolved.
